# Divergence and convergence: a cross-generational study on local food consumption

**DOI:** 10.1038/s41598-024-64284-1

**Published:** 2024-06-12

**Authors:** Jianhong Chen, Anxin Xu, Decong Tang, Manhua Zheng

**Affiliations:** 1 College of Rural Revitalization, Fujian Agriculture and Forestry University, Shangxiadian Street 15, Fuzhou, 350002 Fujian China; 2 College of Journalism and Communication, Minjiang University, Xiyuangong Road 200, Fuzhou, 350108 Fujian China; 3 College of Economics and Management, Fujian Agriculture and Forestry University, Shangxiadian Street 15, Fuzhou, 350002 Fujian China

**Keywords:** Local food, Generation Z, Food choice, Cross-generation, Psychology, Environmental social sciences

## Abstract

In the context of the expanding local food market, grasping the evolutionary trajectory of consumer purchasing behavior is crucial for understanding market dynamics. This study adopts a cross-generational perspective to delve into and elucidate the similarities and differences in local food consumption behaviors between Gen Z and Gen Y. Through the analysis of online survey data from 251 individuals of Gen Z and 319 of Gen Y and utilizing the Theory of Planned Behavior as a theoretical framework, and the study identifies eight key variables. The findings reveal that while Gen Z and Gen Y exhibit a range of common characteristics in their choice of local food,including attention to word of mouth, health consciousness, subjective norms, perceived behavioral control, and attitude.there is a significant divergence in their motivating factors for purchasing. Specifically, convenience is the primary driver for Gen Z when selecting local food; conversely, price is the decisive factor in the decision-making process of Gen Y. By unveiling these significant differences and similarities, the research offers significant understanding beneficial to the food sector, particularly in formulating market strategies targeted at different generations.

## Introduction

With the unprecedented challenges of global climate change, diet-related diseases, and COVID-19, there is a growing concern among the public for the food system’s sustainability^1^. These challenges not only put pressure on the global economy but also pose threats to our ecosystems, health, and societal well-being^2^. In this context, exploring and improving the food system’s sustainability has become an urgent priority. There has been widespread attention and discussion on adopting local production systems to enhance food sustainability in recent years^2^. Research indicates that local food systems have significant advantages in reducing food transportation distances, packaging, and storage requirements, which not only help reduce environmental impact but also promote economic and community sustainability^3^.

Producers, retailers, and marketers have recognized the potential of the local food movement and have increasingly begun to promote locally grown fruits and vegetables to meet the growing consumer demand^4,5^.Consumption of locally sourced food is rapidly gaining popularity as a trend in the food industry, with its potential advantages widely acknowledged^6^.

It is worth noting that the rise of Generation Z (Gen Z) will have a profound impact on the global economy and consumer market. They not only possess significant purchasing power but also shape market demand with unique shopping habits and values^7^. In this process, local food has become an integral part of their daily lives. As Gen Z becomes more focused on sustainability and conscious development, local food is not just an option; it is also an active support for local communities and the environment. For Gen Y, their shopping decisions are also influenced by sustainability and tend to support local production and the community economy. Therefore, local food is not only the consumption choice of Gen Z but also part of Gen Y’s quest for a more meaningful and contributing lifestyle. In this context, by gaining insight into the common preferences of Gen Z and Gen Y for local food and the potential differences, we can obtain a fuller understanding of the psychological mechanisms behind local food consumption. This will help retailers, manufacturers, and decision-makers more accurately understand the needs of different groups and develop market strategies accordingly. However, while some existing studies have revealed key driving factors behind consumer decisions for local foods^8^, exploring differences among various consumer groups, especially across generations and specific needs, remains insufficient. This article aims to address the existing research gap, providing the industry with a more comprehensive and in-depth understanding to better meet the diverse demands of contemporary consumers.

This study focuses explicitly on Generations Y and Z, who, as rapidly evolving social forces, possess unique and common characteristics in terms of consumption habits, values, and technology use. This model considers critical variables such as price, health, convenience, and word-of-mouth, utilizing generational theory and the theory of planned behavior as the theoretical framework, combined with Structural Equation Modeling (SEM) methods. The aim is to uncover and predict the underlying factors that influence Gen Y’s and Gen Z’s attitudes and behavioral intentions towards local food.Through in-depth analysis of the consumption characteristics of Generation Y and Generation Z, the focus of this study is to delve deeper into the reasons that lead them to prefer local foods, identify their common and distinctive characteristics, and thereby support more effective marketing strategies and policy formulation to promote more sustainable consumption patterns.

In summary, by introducing and validating the impact of generational differences on attitudes and behaviors towards local food consumption, our results are anticipated to offer valuable perspectives for retailers and manufacturers on how to better position products and marketing strategies. Additionally, it offers empirical support for policymakers on promoting more sustainable consumption patterns through educational and promotional activities.

## Literature review and hypotheses

### Generation

The concept of generations is widely applied to understand differences between different age groups, and this idea has been thoroughly examined in the research by^9^. People who went through comparable economic and social circumstances in their formative years tend to share similar values, perspectives, preferences, and anticipations. These factors can influence their shopping preferences and behavior, as mentioned in the research by^10^. Therefore, marketers must understand the similarities and differences between different generations to better target their products and services and attract specific audiences^11^.

In previous research, there has been no consistent standard for the exact delineation of the Millennial generation (Gen Y) and Gen Z.While views on the starting years of each generation differ, there is a widespread consensus on the historical and social factors that have shaped the development of these generations^12^. This study refers to the research by^13^ and defines Gen Y as individuals born between 1980 and 1994 and Gen Z as those born after 1995.

#### Food consumption characteristics of Gen Z

Gen Z exhibits a strong sense of exploration and a keen enthusiasm for trying various new foods, being curious about different types of food and flavors, particularly favoring those that are sustainable, organic, and locally sourced^14^. They have a preference for convenience and often engage in online shopping and food delivery, a trend expected to continue to grow^11^. Growing up in the digital age, social media holds great importance for them, and they actively use it to update their digital personas, encourage customers to tag restaurants they like, and actively participate in comment interactions^15^. Furthermore, approximately half of Gen Z expresses a willingness to spend more on healthier foods, emphasizing their concern for food quality and health. These characteristics reflect their modern lifestyle and values, which have a positive impact on the food industry^16^.But due to a lack of skills or environmental or economic constraints, this behavior may not end up happening.^17^

#### Food consumption characteristics of Gen Y

They consider themselves food enthusiasts and place a high emphasis on food quality. When purchasing food, their primary considerations are cost, nutritional value, and the presence of artificial additives^18^.Gen Y say friends and family are their main sources of nutrition information^19^. They tend to prefer options that are both economical and nutritious. While organic and plant-based foods are part of their choices, they are not as crucial as economic and nutritional advantages. Gen Y are more engaged with food and tend to be more health conscious and eat healthier compared to other generations^20^.These characteristics reflect the high attention that Gen Y pays to food.

### Local food

**Figure 1:**
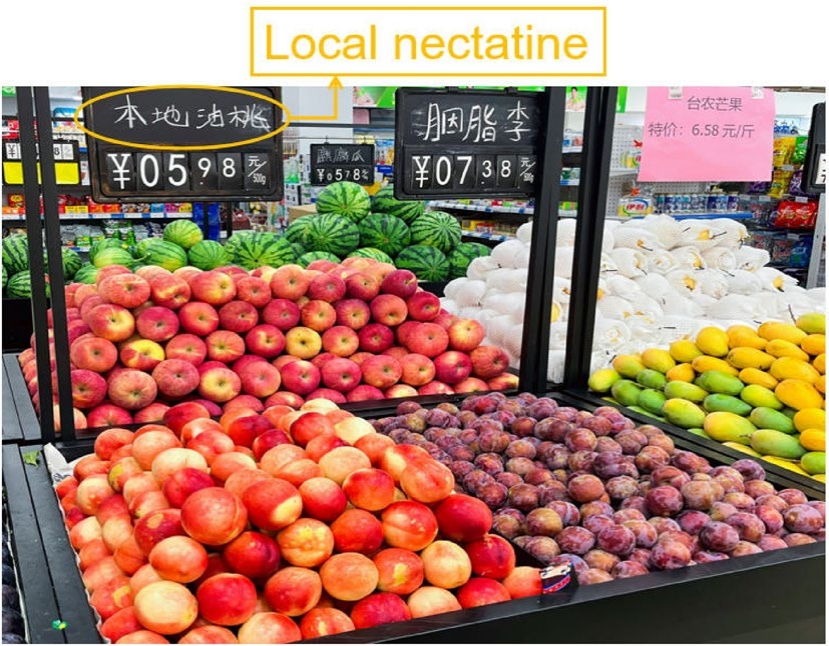
local food.

Despite the increasing attention towards local food (see Fig. 1), the term “local” still lacks a unified definition. Many retailers and producers define local food based on different criteria, including geographic proximity^21^, geographical boundaries^22^, regional characteristics^23^, or food associated with emotions and interpersonal relationships^5^. Regardless of how producers, retailers, or marketers define it, the success of products in the market largely depends on consumers’ acceptance^4^. Therefore, in this context, this study aims to guide consumers in developing their own understanding of “local food,” which may encompass factors such as distance (ranging from 30 to 100 miles), geographical boundaries (national, provincial, or city), and regional specialty foods, among others.

### Motivations for local food choices

Several fundamental factors drive consumers to opt for local food. Primarily, numerous research highlights that local food is believed to possess superior quality, with attributes like freshness, health benefits, and flavor, all of which enhance consumer well-being. Additionally, consumers’ concerns about food safety and transparency are also considered essential non-material factors driving their preference for local food^8,24,25^.

Secondly, some consumers also perceive local food as having organic characteristics. Local food is typically seen as more reasonably priced than organic food. This factor plays a role in motivating people to choose local food^5,26^.

Furthermore, local food consumption is steadily growing due to its convenience and multifaceted benefits. Food choices are a complex process, and we have selected three critical variables from Food Choice Questionnaires(FCQ)^27^ to measure individuals’ influencing factors and priorities in food selection: health, convenience, and price. Food choice questionnaires have been widely validated and used to assess the factors and priorities influencing individual dietary choices^28^. Taking into consideration the characteristics of Gen Y and Gen Z, who increasingly rely on social media and other sharing platforms as their primary sources of information^29^, word-of-mouth is included as an additional variable, providing a more comprehensive assessment of attitudes towards local food within the framework of the Theory of Planned Behavior (TPB).

#### Word of mouth,attitude toward local food

Historically, word of mouth(WOM) has been recognized as a critical factor in consumer decision-making processes. It serves not only as a primary channel for disseminating information about products and services but also significantly influences consumers’ willingness to purchase and their attitudes^30^. Generation Z, in particular, prioritizes product quality and content marketing, relying on others’ purchasing experiences and word of mouth to make informed buying decisions. They place a high value on the effectiveness of content marketing^31^.

As true digital natives who grew up during the age of smartphones and social media, Generation Z is perpetually connected. They are more likely to share and receive recommendations and reviews about local food via social media platforms, influencer endorsements, and interactions with peers^32^. Additionally, Generation Z is more inclined to share their own experiences, thereby influencing others^33^ .

While studies show that Generation Y (Millennials) tends to share their satisfaction with products and services and make recommendations to friends and relatives^34^, their engagement with word of mouth differs from that of Generation Z. Generation Y typically engages in more traditional sharing within their immediate social circles, often prioritizing personal experiences over broader social media influences. This approach contrasts with Generation Z’s extensive use of digital platforms to disseminate and gather information. Consequently, while word of mouth is important to both generations, it has a more decisive and far-reaching impact on Generation Z, who utilize and value peer opinions and reviews to a greater extent in their consumer behaviors.Based on this, we propose the following hypothesis:

##### H1

Compared to Generation Y, the influence of word of mouth on attitude towards local food is more positive for Generation Z.

#### Price, attitude toward local food

Price plays a crucial role in consumer purchasing decisions, especially when it comes to buying local food^5^. Research shows that although consumers generally do not consider local food to be expensive^35^, price remains a significant factor affecting its appeal^26^. Literature also confirms that price factors positively influence consumer purchasing attitudes^36^. Generation Z is more conservative with money. They conduct extensive research and comparisons between online and offline shopping channels before making a purchase, seek discounts, and place a higher emphasis on value for money^37^. While local prices are reasonable, Gen Z may look for more economical food options in times of financial pressure or budget constraints^36^. For the generation Y, cost is the primary consideration when purchasing food. If the price of local foods is competitive compared to imported or non-local foods, we believe they would tend to choose local foods^19^. Therefore, prices may affect the attitudes of the two generations towards local food to varying degrees. It is important to note that “price” here refers to consumers’ subjective perception of price, not the actual cost itself. Consumers’ purchasing decisions are often based on their perception of price rather than objective price levels. Therefore, we propose the following hypothesis:

##### H2

Compared to Generation Z, the influence of price on attitude towards local food is more positive for Generation Y.

#### Health, attitude toward local food

Health issues and related motivations are significant drivers for consumers purchasing local foods^38,39^. Local foods, with their health-oriented features such as freshness and purity, significantly enhance consumers’ overall evaluations of food^40^, thereby fostering a positive attitude towards local foods^26^. However, Generation Z, despite recognizing the importance of incorporating fresh fruits and vegetables into their diet, tends to pay less attention to healthy eating and are more inclined to seek delicious and interesting food experiences^17^. At the same time, Generation Z consumes high-sugar and high-fat foods more frequently, as well as more snacks instead of traditional meals. As Generation Z’s independence grows and their exposure to new social groups increases, this may either promote or hinder their consumption of healthy foods^41^ and also affect their choices of local foods.

In contrast, the attitude of Generation Y is quite different. They are willing to pay extra for healthy foods because they place a high value on personal health and well-being^42^. Therefore, products with health benefits and environmental sustainability features, like local foods, are highly attractive to Generation Y consumers. Based on these observations, we propose the following hypothesis:

##### H3

Compared to Generation Z, the influence of health on attitude towards local food is more positive for Generation Y.

#### Convenience, attitude toward local food

Convenience is one of the main drivers of food choices globally, closely linked to reducing the time and effort consumers need to invest in food shopping, preparation, cooking, or post-meal cleaning^43^. This encompasses various aspects, such as the physical distance consumers travel from home to the shopping location, the mode of transportation used, the density of food procurement points in the area, and their operating hours. Additionally, the convenience is exemplified by retailers offering semi-prepared or prepared foods, such as “pre-washed,” “pre-cut,” “pre-mixed,” and “pre-cooked” products, because these significantly reduce the time needed for food^44^.

Literature has demonstrated that convenience is a significant advantage of local food, and its importance is increasingly emphasized^45^. This is particularly true for Generation Z, who exhibit a high pursuit of convenience in their everyday lives, reflected in their shopping and food choices^46^. The younger generation, especially core members like university students within Generation Z^47^, tends to consume convenient and pre-packaged foods, often due to their lack of cooking skills^48^.

Research indicates that convenience is a key factor valued across generations in food selection^49^. However, social changes and technological innovations have enhanced the convenience of food options (including local foods), presenting Generation Z with an increased selection of convenient foods^50^. Compared to Generation Y, Generation Z places a higher priority on convenience during the shopping process, considering it a primary factor.Therefore, we believe that convenience impacts the purchasing decisions of these two generations to varying degrees. Based on the discussion above, we propose the following hypothesis:

##### H4

 Compared to Generation Y, the influence of convenience on attitude towards local food is more positive for Generation Z.

### Theory of planned behavior

The Theory of Planned Behavior (TPB) aims to understand the intention to act on three determinants: attitude, perceived behavioral control, and subjective norm^51^. These three determining factors are widely considered to accurately predict the intention to perform many behaviors^52^ and have also been applied in the context of local food choice^53^.

TPB is frequently used to study the food consumption behavior of Gen Z and Gen Y. For example, normative beliefs, influences, and opinions of family and friends can increase the willingness of college Gen Y to purchase organic products^54^. In the case particularly relevant to Gen Z, control factors may play a crucial role; young people may live with their parents for a longer time compared to previous generations^52^

Finally, attitude is also considered an essential factor in predicting behavioral intention in the context of local food choices. Research indicates a significant correlation between a positive attitude toward local products and consumers’ willingness to purchase. Studies in the local food consumption literature provide ample evidence that attitude positively influences the intention to purchase local food^55^.

#### Subjective norm and attitude

Subjective norms are related to the social pressure associated with specific behaviors and the extent to which individuals should participate^51^ . When consumers are uncertain about the outcomes of certain behaviors, they may seek support from others^56^ Moreover, social pressure can come from various role players, including friends, family, and others in society^57^ . In the context of this survey, subjective norms are understood as the pressure from significant others who believe that consumers should support, are willing to, and prefer purchasing local food. There is evidence indicating that subjective norms are highly correlated with consumers’ willingness to buy when it comes to the choice of local food.^58^.As a result, we offer the following hypothesis:

##### H5

 Subjective norms positively influence consumers’ attitudes toward local food.

#### Subjective norm, Perceived behavioral control, attitude and purchase intention

Perceived behavioral control(PBC), attitude, and Subjective norms(SN) are considered the primary predictive factors of behavioral intention^51^. PBC can be described as an individual’s perceived ability to engage in a specific behavior, attitude represents their evaluation of that purchase behavior (whether positive or negative), and SN stands for perceived social pressure, i.e., whether the opinions of others about the behavior would influence whether they perform it or not. Past research has extensively explored the relationship between the TPB model and the purchase of local food behavior^58^. The results consistently show that attitude is almost always the most relevant predictive factor closely associated with the individual’s intention. Secondly, there is a moderate level of correlation between the other predictive variables .As a result, we propose the following hypothesis:

##### H6

 Subjective norms positively influence consumers’ purchase intentions for local food.

##### H7

Perceived behavioral control positively influences consumers’ purchase intentions toward local food.

##### H8

Attitude positively influences consumers’ purchase intentions toward local food.

#### Extension of the TPB

**Figure 2:**
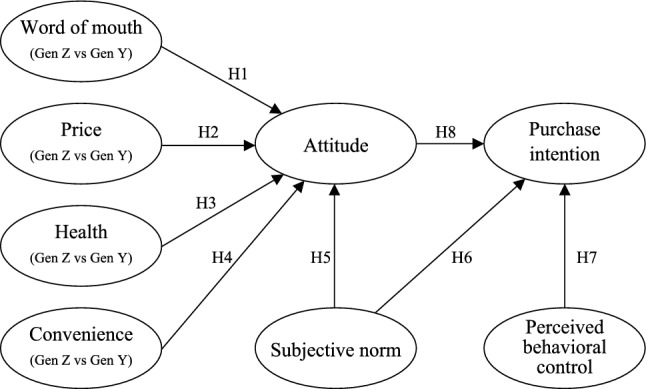
the framework for purchase intention of local food.

Ajzen^51^ is the founder of the Theory of Planned Behavior (TPB), and he has demonstrated that other important intention and behavior prediction variables can be incorporated into the TPB framework. When the intention is related to other significant influencing factors, which play a substantial role in the model’s forecasting accuracy, the model can be expanded. Therefore, the framework of this paper is shown in Figure 2.

## Materials and methods

### Questionnaire

The collection of variables utilised in this study were adapted from prior research and subsequently modified^27,59–61^. Table 1 displays the questionnaire items and their adoption sources. This study examined the peculiarities of the Chinese language and culture. In order to ensure the correctness of the measurements, native speakers were employed to perform back-translation. Every variable was measured using a 7-point Likert scale, extending from 1 (‘strongly disagree’) to 7 (‘strongly concur’). The survey questionnaire has been partitioned into three distinct components. The initial enquiry serves as a screening question. After a brief introduction to local foods, respondents are asked, “What local foods have you purchased recently?” In the final data processing, questionnaires with unanswered inquiries or non-food-related responses will be discarded, leaving only those completed by individuals who purchased local food. The second section of the questionnaire requests interviewees to reminisce about their latest local food consumption experiences and rate their consumer sentiments on a scale. The third stage is to provide individual data, including the respondent’s gender, age, level of education, and income. In addition, the questionnaire contains a screening query to validate the responses. The screening query is placed in the center of the survey to determine whether or not the respondent has provided truthful responses. If an incorrect response to the mathematical query “100+100=?” is given, the survey is terminated.

**Table 1:** Measurement.

Variables	Items	References
Word of mouth	Say positive things about local food to other people.	
	Recommend local food to someone who seeks your advice.	^60^
	Encourage friends and relatives to find out more about local food.	
Price	Is not expensive.	
	Is good value for money.	^27^
	local food price is acceptable	^61^
Health	Keeps me healthy	
	Is good for my skin/teeth/hair/nails,etc	^27^
	Is nutritious	
Convenience	Takes no time to prepare	
	Is easily available in shops and supermarkets	^27^
	Can be bought in shops close to where I live or work	
Attitude	Buying local food would be pleasant.	
	Buying a local food is favourable	^59^
	Buying local food is a wise choice.	
Subjective norm	Most people important to me, think that I should buy local food.	
	Most people, important to me, would want me to purchase local food	^59^
	People whose opinion I value would prefer that I should buy local food.	
Perceived behavioral control	I am confident that if I want, I can buy local food.	
	If I want to, I can easily buy local food	^59^
	To buy or not to buy local food is entirely up to me.	
Purchase intention	I will consider purchasing local food when needed.	
	I am willing to buy local food while shopping.	^59^)
	I will make an effort to buy local food	

### Data collection and Sample

To verify the proposed model, we conducted an online survey on Credamo (https://www.credamo.com). Credamo is a professional measurement platform in China that offers services to numerous researchers and has been demonstrated to be a dependable data collection platform comparable to Amazon Mechanical Turk^62^. Credamo has a more diverse sample pool, which is an obvious advantage. 50 Chinese participants were used to evaluate the questionnaire’s readability and fluency to ensure the validity of the survey. Following initial testing, ambiguous and inappropriate questions were modified. Our survey offered multiple “red envelopes” as incentives. Upon completion of the survey, participants could receive a “red envelope” containing 3 RMB. In order to prevent duplicate survey submissions, each IP address was restricted to a single submission.We asked participants to have an 80% credit score and history acceptance rate, and established age criteria to ensure that only individuals in the Gen Z and Gen Y age groups could complete the questionnaire. The platform managed the questionnaire randomly to ensure a diversified distribution of participants across different provinces in China. After collecting 600 questionnaires, the survey obtained 570 valid responses.

### Ethical approval

Our study was not medical research nor employed any experiments on humans, and we used a survey to collect data. According to the Declaration of Helsinki, our institution deemed ethical approval not required. Furthermore, the gathered information is strictly confidential and anonymous and is only used for research purposes. Informed consent was obtained from all participants and/or their legal guardians.

## Result

### Demographic profile

We analyzed the above data with descriptive statistics applied to jamovi, and the results are presented in the Table 2. Regarding gender distribution, females were the prominent participants in the study 67.9%. Females acted as regular food purchasers in the household. The obtained result aligns with the outcomes reported in other research. It is likely since women tend to be more consumptive than male^63,64^.

**Table 2:** Demographic characteristics of the sample (N=570).

	Items	Frequency (total)	Proportion (total) (%)	Y (N$=$319)	Z (N$=$251)
Gender	Female	387	67.9	200	187
	Male	183	32.1	119	64
Education	High school and below	74	12.9	42	32
	College	405	71.1	214	191
	Undergraduate	83	14.6	56	27
	Postgraduate and above	8	1.4	7	1
Monthly income (RMB/Yuan)	Below 2000	71	12.5	2	69
	2000-4000	72	12.6	10	62
	4000-6000	107	18.8	54	53
	6000-8000	99	17.4	69	30
	8000 and above	221	38.8	184	37

As a focal point of this study, respondents were divided into two age groups: Gen Y and Gen Z. Both groups consisted of approximately 300 participants to ensure fairness, consistent with previous literature on generational differences^65^. Consumers were further segmented based on age groups, and the sample size was sufficient to acquire reliable results^17,66^.

Moreover, a significant proportion of respondents had a high level of education, with over 71.1% possessing an undergraduate degree. This advanced degree of schooling aligns with previous research findings^26^. In terms of monthly income, more than 38.8% of individuals reported an income of 8000 or above. These data are consistent with previous studies on the consumption of local food. In general, the study’s sample matched the demographics of similar studies conducted on local food in China.^67^.

### Independent samples t-test

**Figure 3:**
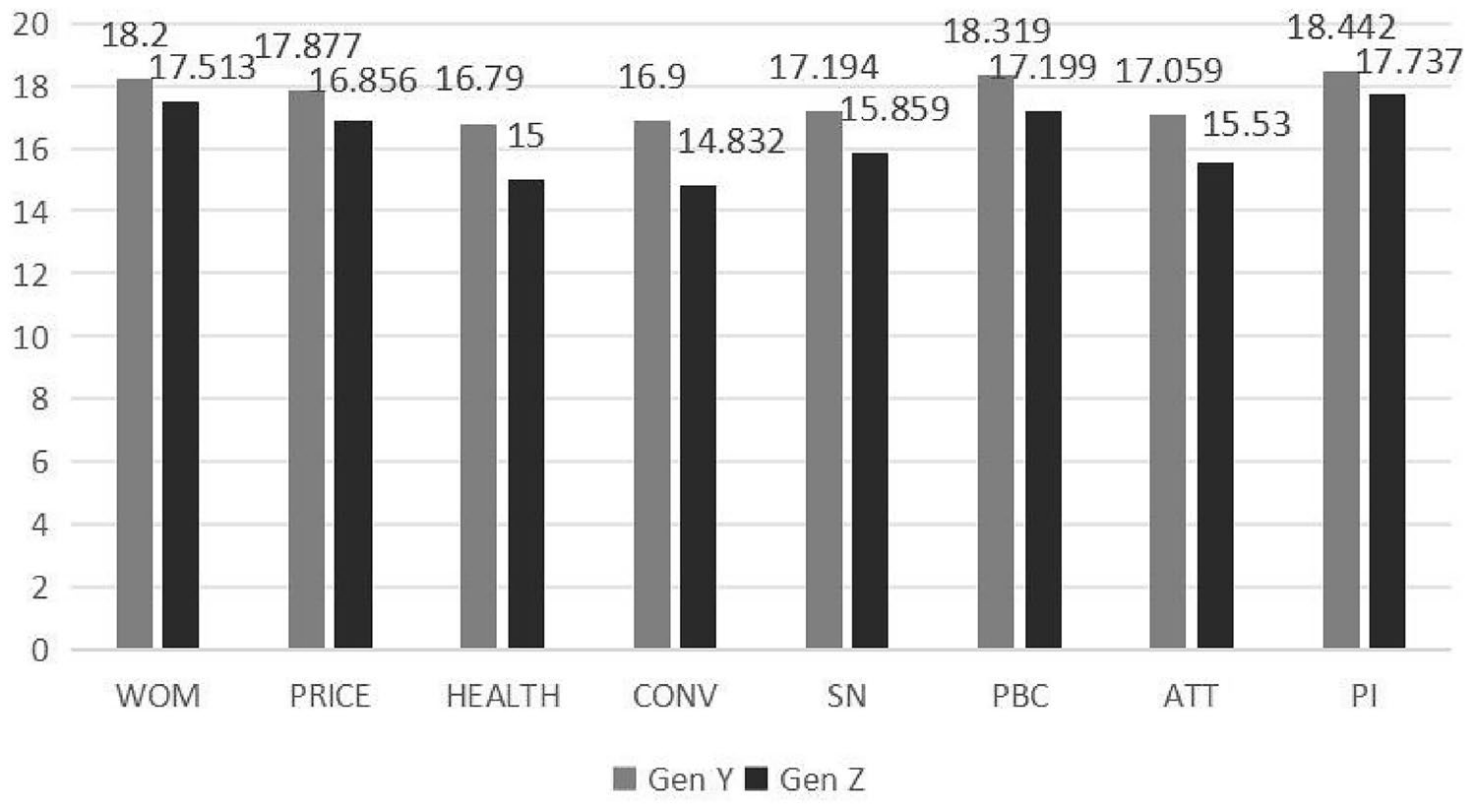
Mean value of all variables.

The analysis of the data presented in Table 3 and Fig. 3 reveals discernible variations across eight characteristics between those belonging to Gen Z and those who do not.: word of mouth, price, convenience, health, subjective norms, perceived behavioral control, attitude, and purchase intention. The *p*-values for all eight variables exhibit statistical significance, as they are all below the threshold of 0.01.

**Table 3:** Independent samples t-test.

Constructs	Mean ± S.D. (Gen Y)	Mean ± S.D. (Gen Z)	Value	Sig.
WOM	18.200 ± 1.979	17.513 ± 2.662	3.411	0
PRICE	17.877 ± 2.185	16.856 ± 3.104	4.421	0
HEALTH	16.790 ± 3.030	15.000 ± 3.445	6.489	0
CONV	16.900 ± 3.081	14.832 ± 4.103	6.642	0
SN	17.194 ± 2.948	15.859 ± 3.479	4.847	0
PBC	18.319 ± 2.000	17.199 ± 2.939	5.171	0
ATT	17.059 ± 2.514	15.530 ± 3.221	6.185	0
PI	18.442 ± 1.816	17.737 ± 2.341	3.930	0

Specifically, Gen Y generally scored higher than Gen Z on these variables, suggesting that the former group had a more favorable evaluation of local food. In particular, the disparities between Gen Z and Gen Y were particularly evident regarding subjective norms and attitudes.

These findings further substantiate the necessity of investigating the disparities between the two cohorts and establishing a foundation for subsequent econometric examination. In the subsequent section, we will explore the distinct disparities between those two groups in relation to local food consumption, with the aim of formulating more complete conclusions.

### Reliability and validity analysis

The research employed the use of smart-pls as a means to address the application of PLS-SEM. This choice was made owing to the primary focus of the analysis on examining the correlation between variables, as opposed to structural equation modelling. By utilising smart-pls, the model was able to assess the viability of all pathway coefficients, leading to the development of a more resilient and reliable model. In this work, three reliability metrics were utilised, namely Cronbach’s Alpha, Rho_A, and Composite Reliability. The results are displayed in Table 4, and the values exceed the respective critical thresholds of 0.6, 0.7, and 0.8, indicating the reliability of the scale.^68^. The validity of the scale was then evaluated. As displayed in Table 4, all constructs had AVEs over 0.5, indicating a variance greater than 50% and acceptable convergent validity^69^. As demonstrated in Table 5, all external factor loadings were higher than 0.7, showing that all scale items had positive external loadings^70^. FORNELL and HTMT(seen in Tables 6 and 7) were used to measure the discriminant validity. As noted in Table 4, the square root of AVEs is greater than the absolute value of the Pearson correlation coefficient of each variable. The provided evidence serves as an initial demonstration of strong discriminant validity.^69^, and the current study’s findings meet this requirement. The HTMT between the two constructs was less than 0.90, further demonstrating good discriminant validity^68^ and offering indirect evidence that the data were not multicollinear^71^.

**Table 4:** Reliability and validity analysis.

Constructs	Cronbach’s Alpha	rho_A	Composite reliability	AVE
Attitude	0.737	0.752	0.850	0.655
Convenience	0.761	0.829	0.856	0.667
Health	0.854	0.857	0.911	0.774
Perceived behavioral control	0.716	0.717	0.841	0.638
Purchase intention	0.701	0.711	0.833	0.625
Price	0.787	0.795	0.875	0.700
Subjective norm	0.869	0.871	0.919	0.792
Word of mouth	0.739	0.752	0.850	0.655

**Table 5:** Factor loadings and VIF.

Constructs	Indicator	Indicator reliability	VIF
Attitude	ATT_1	0.782	1.380
	ATT_2	0.774	1.469
	ATT_3	0.868	1.663
Convenience	CON_1	0.861	1.436
	CON_2	0.854	1.970
	CON_3	0.728	1.618
Health	HEALTH_1	0.889	2.206
	HEALTH_2	0.863	2.005
	HEALTH_3	0.889	2.164
Perceived behavioral control	PBC_1	0.769	1.331
	PBC_2	0.822	1.527
	PBC_3	0.804	1.414
Purchase intention	PI_1	0.750	1.350
	PI_2	0.813	1.332
	PI_3	0.807	1.445
Price	PRICE_1	0.832	1.734
	PRICE_2	0.858	1.661
	PRICE_3	0.820	1.572
Subjective norm	SN_1	0.898	2.296
	SN_2	0.890	2.338
	SN_3	0.883	2.224
Word of mouth	WOM_1	0.802	1.514
	WOM_2	0.781	1.419
	WOM_3	0.844	1.471

**Table 6:** Discriminant validity (FORNELL).

	(1)	(2)	(3)	(4)	(5)	(6)	(7)	(8)
Attitude (1)	0.810							
Convenience (2)	0.450	0.816						
Health (3)	0.666	0.466	0.880					
Perceived behavioral control (4)	0.403	0.435	0.373	0.799				
Purchase intention (5)	0.567	0.334	0.444	0.484	0.790			
Price (6)	0.478	0.375	0.385	0.380	0.511	0.837		
Subjective norm (7)	0.659	0.434	0.668	0.380	0.427	0.389	0.890	
Word of mouth (8)	0.586	0.381	0.509	0.373	0.598	0.505	0.528	0.809

**Table 7:** Discriminant validity (HTMT).

	(1)	(2)	(3)	(4)	(5)	(6)	(7)	(8)
Attitude (1)								
Convenience (2)	0.560							
Health (3)	0.837	0.555						
Perceived behavioral control (4)	0.557	0.595	0.478					
Purchase intention(5)	0.767	0.457	0.565	0.685				
Price (6)	0.614	0.478	0.465	0.506	0.683			
Subjective norm (7)	0.817	0.507	0.774	0.484	0.537	0.467		
Word of mouth (8)	0.773	0.515	0.634	0.514	0.832	0.657	0.651	

### Assessment of structural model

Tables 8 and 9 present the results of the model evaluation applying a bootstrapping subsamples 5000 research. The variance inflation factor (VIF) was used to measure the common method bias^72^, when the variance inflation factor (VIF) is less than the crucial value of 5,there is no common method bias in the study. According to R2, all constructs are greater above the critical value of 0.25, demonstrating the model’s strong ability to forecast the future^73^. Indicative of out-of-sample predictive relationship and medium forecasting power are all constructs larger than 0.15 and greater than Q2^74^. The model fitting index SRMR is 0.065 < 0.080, it indicates that the model fit is superior^75^.

**Table 8:** Internal model VIF values.

	(1)	(2)	(3)	(4)	(5)	(6)	(7)	(8)
Attitude (1)					1.856			
Convenience (2)	1.393							
Health (3)	2.034							
Perceived behavioral control (4)					1.228			
Purchase intention (5)								
Price (6)	1.4382							
Subjective norm (7)	2.026				1.82			
Word of mouth (8)	1.700

**Table 9:** Model results.

	$R^2$	$Q^2$	SRMR
Attitude	0.590	0.377	0.065
Purchase intention	0.400	0.242	

### Structural equation modeling

To test the model, 5000 bootstraps were run using PLS-SEM. The results are displayed in Table 10. Word of mouth positively influences Gen Z’s perception of local food, according to the data from the Table 10 ($\beta$=0.280; $p<$ 0.001), and it also positively affects Gen Y’s attitude towards local food ($\beta$ = 0.131; $p<$ 0.05), unsupporting H1.Price does not affect Gen Z’s attitude towards local food ($\beta$= 0.025; $p>$ 0.1). However, it positively affects the attitudes of Gen Y towards local food ($\beta$= 0.286; $p<$ 0.05), supporting H2. Health significantly affects Gen Z’s attitudes toward local food ($\beta$= 0.272; $p<$ 0.001), and it also positively affects Gen Y ($\beta$= 0.280; $p<$ 0.001), unsupporting H3. Convenience significantly positively affects Gen Z’s attitude toward local food ($\beta$= 0.101; $p<$ 0.5). However, for Gen Y, this does not affect their attitude towards local food ($\beta = -0.036$; $p>$ 0.1), supporting H4. Subjective norm does not impact on consumers purchase intention of local food ($\beta$=0.034; *p* =0.577> 0.1) and crosses 0 within the 95% confidence interval, unsupporting H5. Subjective norm favourable affects consumers attitude toward local food ($\beta$= 0.273; $p<$ 0.001), supporting H6. Perceived behavioral control has a favourable influence on consumer’s intention to purchase local food ($\beta$ = 0.300; $p<$ 0.001), therefore supporting H7. Attitude has a significant and positive effect on consumers’ intention to purchase local produce ($\beta$ = 0.423; *p* < 0.001); therefore, H8 is supported.

**Table 10:** Hypothesis testing.

Hypotheses	$\beta$	95%LLCI	95%ULCI	Mean	SD	t-Value	*p*-Value
WOM$\rightarrow$ ATT (Gen Z)	0.280	0.132	0.408	0.274	0.084	3.344	0
WOM $\rightarrow$ ATT (Gen Y)	0.131	0.033	0.216	0.126	0.056	2.32	0.02
PRICE $\rightarrow$ATT (Gen Z)	0.025	− 0.06	0.119	0.027	0.055	0.456	0.648
PRICE$\rightarrow$ ATT (Gen Y)	0.286	0.193	0.371	0.282	0.054	5.335	0
HEALTH$\rightarrow$ ATT (Gen Z)	0.272	0.167	0.381	0.274	0.065	4.181	0
HEALTH$\rightarrow$ ATT (Gen Y)	0.280	0.191	0.371	0.281	0.055	5.118	0
CON$\rightarrow$ ATT (Gen Z)	0.101	0.021	0.187	0.106	0.050	2.033	0.042
CON$\rightarrow$ ATT (Gen Y)	− 0.036	− 0.109	0.053	− 0.028	0.049	0.732	0.464
H5:SN$\rightarrow$ PI	0.034	− 0.063	0.139	0.037	0.061	0.557	0.57
H6:SN$\rightarrow$ ATT	0.273	0.186	0.36	0.274	0.053	5.144	0
H7:PBC$\rightarrow$ PI	0.300	0.208	0.394	0.303	0.056	5.345	0
H8:ATT$\rightarrow$ PI	0.423	0.318	0.518	0.421	0.061	6.942	0

### Multiple-group analysis

To determine whether there are differences in the influencing factors of attitudes and intentions towards local food between the two groups, we conducted analyses for both Gen Z and Gen Y individuals. Therefore, we used (5000 permutation runs, a two-tailed test, and a significance level of 0.1) to identify the significant differences between Gen Z and Gen Y relationships. Table 11 presents the parametric test results of the multiple-group analysis. Based on our analysis, we found differences between the two groups. Specifically, Price to attitude $p=0< 0.01$, t-value=3.400, and convenience to attitude *p*-value=0.05 < 0.1, t-value=1.95. It suggests differences between the two groups regarding their attitudes towards local food concerning Price and convenience. However, we did not observe any differences between the two groups regarding the influence of word of mouth, health, and subjective norms on attitudes toward local food. Similarly, the two groups had no significant differences regarding the influence of attitude and subjective norms on the intention for local food.

**Table 11:** MGA-parametric test.

	Path Coefficients-diff	t-Value (Gen Y vs Gen Z)	p-Value (Gen Y vs Gen Z)
WOM $\rightarrow$ ATT	− 0.149	1.518	0.130
PRICE $\rightarrow$ ATT	0.261	3.400	0.000
HEALTH $\rightarrow$ ATT	0.008	0.093	0.926
CONVENIENCE $\rightarrow$ ATTI	$-0.137$	1.951	0.051
SN $\rightarrow$ PI	0.159	1.421	0.155
SN $\rightarrow$ ATT	0.067	0.633	0.527
PBC $\rightarrow$ PI	0.104	1.052	0.293
ATT $\rightarrow$ PI	− 0.037	0.344	0.731

**Figure 4:**
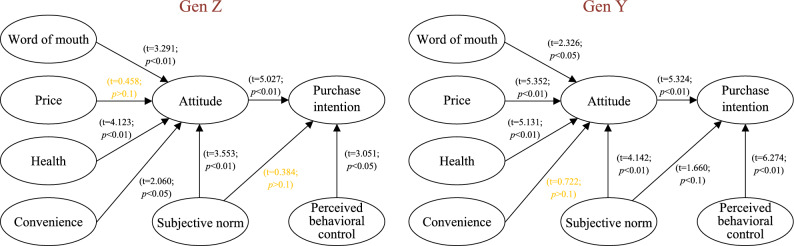
MGA result.

Above, we conducted a combined analysis of Gen Z and Gen Y groups as a single entity. In Fig. 4, we individually compare the attitudes and intentions of Gen Z and Gen Y groups towards local food.

For Gen Z, the influence of price on the attitude they have yielded a *p*-value greater than 0.1, with a t-value of 0.458. It suggests that price does not significantly affect the attitudes of Gen Z toward local food. Additionally, the influence of subjective norms on purchase intention also resulted in a *p*-value greater than 0.1, with a t-value of 0.384, indicating that subjective norms do not significantly impact the purchase intention of Gen Z towards local food. On the other hand, word of mouth, health, convenience, and subjective norms influence Gen Z’s attitudes toward local food. Furthermore, attitudes and perceived behavioral control impact Gen Z’s intentions toward local food.

For Gen Y group, the influence of convenience on attitude yielded a *p*-value greater than 0.1, with a t-value of 0.772. Similarly, this indicates that convenience does not significantly affect the attitudes of the Gen Y group towards local food. Word of mouth, price, health, and subjective norms do influence the attitudes of the Gen Y group toward local food. Additionally, attitudes, subjective norms, and perceived behavioral control impact the intentions of the Gen Y group toward local food.

## Conclusions, discussion and management implications

### Conclusions

The results of this study are discussed with reference to the 8 hypotheses proposed. For hypothesis H1, we found that word of mouth is a key factor that equally influences both Generation Z and Generation Y in their choice of local food. This may be due to a few reasons: On one hand, it is closely related to the environments in which both generations have been raised, as they grew up with the internet and are reliant on social media, making decisions based on reviews^76,77^. On the other hand, Generation Y might feel external pressures, particularly from social media influencers and friends, which can influence their purchasing behaviors. Additionally, as access to product information increases, the expectations of Gen Y regarding products and services also rise, making them more likely to share their experiences^78^, contributing to a robust feedback and information dissemination cycle. Therefore, this contradicts our previous hypothesis H1.

Regarding Hypothesis H2, this aligns with our assumption that Generation Z tends to pursue hedonism, which means when it comes to choosing food, they prioritize enjoyment and unique experiences over price. This preference reflects their focus on immediate gratification and sensory experiences rather than cost-effectiveness^79^. Therefore, although Generation Z may be sensitive to price, their purchasing decisions are more likely to be influenced by the novelty of the experience or the uniqueness of the product, rather than merely the price. In contrast, Generation Y may place more emphasis on value for money in their everyday consumption^80^. The appeal of local food for Generation Y may stem from its reasonable pricing and high quality or support for local communities. This focus on cost-effectiveness leads Generation Y to consider price factors more when purchasing local food, viewing it as an economical choice and a responsible consumption practice. Thus, the consumer tendencies of Generation Y may lead to a different response to price factors compared to Generation Z.

According to hypothesis H3, health is a common factor influencing both generations’ choice of local food. One reason is that this survey was conducted against the backdrop of a global pandemic. This context may lead people to associate the survey results with the aftermath of the pandemic, as there is an increased focus on personal health issues^28^. Therefore, both generations are more inclined to choose local foods that enhance health. Another reason is that research^81^ shows that young people tend to engage in healthy behaviors (such as exercising and increasing fruit and vegetable consumption) as a form of compensation for unhealthy habits related to excessive drinking and fast food/candy diets. Therefore, Generation Z is willing to choose healthy local foods to compensate for unhealthy lifestyle behaviours.

Turning to H4, a difference was found between the two generations regarding convenience, which is consistent with our initial hypothesis Gen Z and Gen Y may have different lifestyles and habits, leading to different responses to convenience. For Gen Z, convenience influences their attitude towards local food because they are representatives of the digital age, more skilled in using smartphones, social media, and online applications. This makes it easier for them to find, purchase, and enjoy local food. The need for convenience is more apparent in services like online ordering, takeout, and food delivery^82^, aligning with the more digital lifestyle of Gen Z^83^. For Gen Y, convenience does not affect their choice of local food, possibly because they value the price of food more than convenience. Gen Y may rely more on traditional shopping methods, which might make convenience less influential on their attitudes.

H5 and H6 are discussed together. Subjective norms influence attitudes towards purchasing local food but not the intention to purchase it. This might indicate a discrepancy or conflict between attitudes and actual behavior. Here are some possible explanations: Subjective norms might affect individuals’ attitudes towards local food because they feel societal or peer expectations. They may believe that purchasing local food is popular and aligns with social norms, leading to a supportive attitude towards local food. However, while their attitudes are influenced by societal norms, their actual purchase intentions might be constrained by other factors, such as price, availability, and actual needs. Even if they have a positive attitude towards local food, they might still be reluctant to purchase if they face inconvenience or high prices.

Finally, H7 and H8 suggest that perceived behavioral control and attitudes influence the purchase intentions of both generations towards local food. Based on the discussion above, it’s believed that purchasing local food is valuable and meets the needs of these two generations, making them more likely to take purchasing actions. Previous literature also sufficiently demonstrates the positive and significant influence of perceived behavioral control and attitudes on purchase intentions^58^.

In conclusion, this study sheds light on the intricate dynamics of food choice behaviors in Gen Z and Gen Y, offering valuable insights for marketers and policymakers aiming to understand and influence these demographic segments. The findings underscore the significance of word of mouth, convenience, price, and health.In our exploration, we discovered commonalities; however, what’s more important is the identification of differences. Notably,the differences between the two generations in their local food choices, with convenience being a predominant factor for Gen Z, whereas price holds more importance for Gen Y. This nuanced understanding of generational preferences is crucial for effectively targeting and catering to the distinct needs and behaviors of these two influential consumer groups.

### Discussion

The following discussion is presented to explore further the reasons behind the significant differences between Generation Z and Generation Y in their evaluation of local food.

The impact of price on the attitudes of both generations towards local food may stem from various reasons. For Generation Z, being the youngest consumer group and typically at the beginning of their career paths, their financial resources are limited^84^. This makes them particularly sensitive to price and inclined to seek high-value food deals and promotional offers to extend the value of every money spent^11^. Moreover, Generation Z is more likely to access and accept technologically produced alternative foods, such as lab-grown meat or functional foods, which are often more cost-effective and align with their preferences for novelty and fun^85^. Research has shown that college students prioritize enjoyment and taste over health when choosing foods, simply because they perceive unhealthy foods to taste better, be cheaper, or more accessible^86^. Therefore, despite the acceptable pricing of local foods, they are more likely to opt for other foods based on price perception. In contrast, Generation Y tend to seek products that offer quality and reasonable pricing in their everyday consumption^87^. Thus, local foods’ reasonable pricing attributes make them a preferred choice for Generation Y.

Regarding the differences in convenience, first, it may be related to food nostalgia. Generation Y may associate convenience food with family, traditional cooking habits, and rural life. This nostalgic feeling may incline them to prepare food from scratch rather than opting for convenience foods^88^. This is linked to the less widespread availability of convenience foods during their upbringing than today. In contrast, Generation Z grew up in an era of advanced technology and readily available convenience foods. Their food nostalgia is likely tied to industrialized foods, more integrated with social activities and modern lifestyles^43^. Therefore, Generation Z is more accepting of and prefers convenience foods.

Secondly, the food choices of Generation Z are more diverse. With the rapid growth of global supermarkets and retail chains, Generation Z faces a broader array of food options, including many convenience foods^89^. This variety may make Generation Z more inclined to utilize convenience foods to cope with busy lifestyles and work pressures, even if their cooking skills have improved.

Thirdly, time pressure and a lifestyle of juggling multiple responsibilities are characteristics of modern society, particularly affecting Generation Z^88^. This lifestyle necessitates reliance on quick, convenient food solutions, increasing the demand and consumption of convenience foods^43^. Convenience influences food choices and reflects broader social and economic trends, such as the importance of time management and quality of life.

The differences between the generations are inevitable, as Generation Z has adeptly navigated numerous significant changes in politics, society, technology, and the economy throughout their brief lifetime^90^. The behavioral patterns of Generation Z differ from the previous generation, as extensively documented in the literature^91^. Understanding these differences allows stakeholders like retailers, manufacturers, and policymakers to more effectively promote the sustainable development of local foods, while considering the unique needs and preferences of different generations in food choices.

### Theoretical contribution

The current study makes a valuable contribution to the extant academic literature by addressing several gaps in previous studies. Firstly, some studies have analyzed aspects of food behavior among specific generations^7,17,92^, but there is a noticeable lack of research aimed at analyzing generational differences. In addition, this study employed a generational cohort strategy and explored the overall behavioral intentions of Gen Z and Gen Y rather than focusing solely on specific food choices^93^. This novel perspective provides new insights into generational cohort research and addresses the call for this approach in food. Simultaneously, we sincerely call for more scholars to join the field of generational research. Secondly, Gen Z has already significantly impacted society and the economy^94^ Gen Z Food Trends & Eating Habits, but research on Gen Z in marketing literature is limited. Cohort studies show that different generations often exhibit significant behavioral differences^95^. Thus, targeted marketing strategies are needed. Previous marketing literature has focused on Gen Z’s media consumption^96^, online behavior^97^, and other aspects. At the same time, this paper enriches food marketing research related to Gen Z. Additionally, other behaviors of Gen Z await exploration by more scholars. Furthermore, this paper enriches existing food marketing research related to Gen Z by comparing their local food attitudes with those of Gen Y individuals. The study considers respondents from Gen Z and Gen Y, providing a new generational research perspective and expanding the scope and possibilities of generational research. This further segments consumer groups and creates more research space for scholars.

### Management implications

Our findings have important managerial implications for food retailers and suppliers targeting Gen Z and Gen Y shoppers. In order to increase sales of local food products and increase revenues, retailers and suppliers^98^ must successfully translate and apply new knowledge about changing customer behaviors and evolving needs and incorporate it into their communications with consumers (cf.^99,100^.

Thus, retail managers and marketers need to closely monitor consumers’ shopping behavior and shopping preferences to determine what strategies need to be employed^101^. However, consumer motivation and purchase engagement is often hidden beneath the surface of age; by considering generational cohorts, we can gain a deeper understanding. Generational cohort theory has become a useful tool for segmenting the marketing market, so based on the findings of this paper, retailers and marketers must take a different approach to effectively segmenting these two generational groups.

On the one hand, food retailers need to recognize that Gen Z consumers focus on convenience when choosing local food products, especially those that require no preparation and are easily accessible. Therefore, when targeting Gen Z, it is recommended to increase the convenience of local food products, such as increasing the proportion of ready-to-eat food products, to meet the needs of those Gen Z consumers who do not need to prepare and can eat without preparation. This includes convenient packaging, portioning, or small portion sizes so that consumers can enjoy them anytime, anywhere to make them more appealing and thus increase Gen Z’s willingness to buy.

On the other hand, Gen Y consumers are more price-conscious about local food products and are more likely to accept local food products that are affordable and offer value for money. Therefore, the marketing strategy for Gen Y should focus on offering competitive prices, such as understanding the price sensitivity of the target market, formulating a reasonable pricing strategy and emphasizing the value for money of local foods.

### Research limitations and future research

This study has certain limitations. First, although this research provides empirical insights into the psychological factors influencing different generations’ preferences for local food, it used an FCM questionnaire and selected only three variables. Subsequent inquiries into local food consumption should incorporate additional variables, and we recognize that the decision-making process regarding food choices is multifaceted. It not only hinges on the sensory attributes of food (such as flavor, aroma, and texture) but is also shaped by a spectrum of non-sensory factors. These non-sensory factors include familiarity with the food, expectations of the food, ethical issues, and emotions^102^. Culture, economic level, and policy regulations can provide more perspectives. For example, understanding the factors influencing consumer food choices in specific cultures is key to successfully developing products, as many consumers are dedicated to comparing differences in food preferences and descriptions across different cultures. Perhaps, in cultures that emphasize tradition and local food, both generations might hold a similar positive attitude towards local food, and the differences between Gen Y and Gen Z may diminish or even disappear. After all, people from specific cultures tend to prefer the type of food they are accustomed to eating within their culture, as these foods are readily available^103^.

Secondly, the differences between Gen Y and Gen Z regarding local food in terms of convenience and price may be related to changes in consumption expenditure, life experiences, technological development, and environmental issues. Individuals born in the same period and raised through similar experiences will have similar values, attitudes, beliefs, and expectations, which remain unchanged throughout a generation’s lifetime and constitute a generational identity. In the consumer environment, generational identity significantly influences purchasing patterns and shopping behavior^10^.^76^ adds that because they possess a collective personality and share similar life paths, they tend to share a wide range of values and characteristics. Therefore, Gen Y and Gen Z have different shopping and purchasing goals. These behaviors and purchasing actions are unique to their generation and differ from other generational groups that hold different values^104^.

In addition, the theoretical model presented in this paper can be extended by implementing it in more representative groups in different countries, such as Generation X, to explore differences in inter-generational food choices further.Cross-cultural research is fundamental as it allows for comparisons between countries, which can help formulate marketing strategies for the global food market.

Current research reveals factors influencing Gen Y and Z’s attitudes toward local food. To better explain the differences between the two generations in their approach to local food, future research could consider introducing factors such as environmental awareness, food loyalty, values, and lifestyles, as these could lead to different understandings and degrees of identification with the concept of local food by the two generations. It would help provide more targeted insights and recommendations for relevant stakeholders (such as retailers, manufacturers, policymakers, etc.) to promote the sustainable development of local food.

## Data Availability

The datasets generated during and/or analysed during the current study are available from the corresponding author on reasonable request
